# Detection of Severe Acute Respiratory Syndrome Coronavirus 2 in Pregnant Women Treated with Nirmatrelvir/Ritonavir (Paxlovid) Using Salivary Polymerase Chain Reaction: A Prospective Cohort Study

**DOI:** 10.3390/microorganisms12122566

**Published:** 2024-12-12

**Authors:** Chun-Han Tseng, Chih-Wei Lin, Pei-Yin Tsai, Mei-Tsz Su

**Affiliations:** Department of Obstetrics and Gynecology, National Cheng Kung University Hospital, College of Medicine, National Cheng Kung University, 138 Sheng-Li Road, Tainan 704, Taiwan; lovecassiopeia@gmail.com (C.-H.T.); yowchi66@hotmail.com (C.-W.L.)

**Keywords:** severe acute respiratory syndrome coronavirus 2, pregnancy, nirmatrelvir/ritonavir (Paxlovid), saliva

## Abstract

Objectives: We aim to study the relative viral load using salivary polymerase chain reaction among pregnant women treated with Paxlovid. Methods: Pregnant women with coronavirus disease 2019 were allocated to two groups: those receiving Paxlovid and those receiving no antiviral agents. We compared the nasopharyngeal and salivary relative viral loads and their changes in saliva specimens. Results: Among the thirty-seven pregnant women, seventeen received Paxlovid, and twenty received no antiviral agents. The viral cycle threshold value of saliva was significantly higher than that from nasopharynx, with a median ± interquartile range of 26.44 ± 7.68 versus 17.6 ± 9.6 in the Paxlovid group (*p* = 0.005). Following treatment, the median salivary viral load decreased by 13.40 cycle threshold values in the Paxlovid group (from a median of [Day 0 Ct] to [Day 4/5 Ct]), compared to a change of −1.59 cycle threshold values in the no-antiviral group (from a median of [Day 0 Ct] to [Day 4/5 Ct]) (*p* = 0.021). The detection rate of coronavirus disease 2019 using salivary polymerase chain reaction was 83.8% (31/37). Conclusions: This study showed that saliva is a useful diagnostic tool for coronavirus disease 2019 in pregnant women, and a significant decrease in the relative viral load of saliva was observed in those treated with Paxlovid.

## 1. Introduction 

Coronavirus disease 2019 (COVID-19) continues to pose a significant global health threat and has impacted populations worldwide. Pregnant women are a particularly vulnerable group owing to their susceptibility to infection and potential for adverse outcomes when infected with severe acute respiratory syndrome coronavirus 2 (SARS-CoV-2). Recent research indicates that pregnant women with COVID-19 may experience higher rates of severe illness, an exaggerated immune response, and an increased incidence of preterm birth [1]. These findings underscore the importance of implementing effective measures to protect them from COVID-19 and associated complications.

There is an urgent need for safe and effective oral antiviral treatments that can impede the progression of COVID-19 infection to severe disease, hospitalization, or shorten the time to clinical recovery [2]. Paxlovid is an oral formulation consisting of nirmatrelvir, a potent protease inhibitor, and ritonavir, which functions as a CYP3A inhibitor. It has demonstrated antiviral activity against SARS-CoV-2 and has been shown to lower the risk of hospitalization and death in patients with COVID-19 [3]. Recent research has shown that Paxlovid leads to a lower viral load than placebo when treatment is initiated within 3 days after the onset of symptoms among nonhospitalized, symptomatic adults with high risk for severe disease [4]. However, the effect of Paxlovid on the viral load change is unclear in the pregnant population.

Nasopharyngeal specimens are widely acknowledged as optimal for diagnosing respiratory tract viral infections. However, saliva specimens may serve as viable alternatives because their collection does not require the expertise of a medical professional, thereby minimizing the risk of viral transmission. Previous studies have validated saliva as a diagnostic tool for respiratory viruses compared to nasopharyngeal aspirate (influenza virus and respiratory syncytial virus) [5] or nasopharyngeal swab (influenza virus) [6] using PCR. Furthermore, detection rates for various respiratory viruses have been comparable between saliva and nasopharyngeal swab specimens [7]. Consequently, there has been advocacy for evaluating saliva as the specimen of choice for detecting emerging respiratory viruses [8]. The use of saliva has also been reported for diagnosing COVID-19 and viral load testing [9].

There is currently a dearth of reports on the use of salivary PCR for detecting SARS-59 CoV-2 among pregnant women and studies investigating the use of Paxlovid in pregnant women with COVID-19. Additionally, the association between this population’s antiviral therapy and viral load remains unexplored. Therefore, we examined the utility of self-collected saliva for diagnosing COVID-19 among hospitalized pregnant women and serial viral loads using saliva specimens from patients treated with Paxlovid in the present study.

## 2. Materials & Methods

### 2.1. Clinical Characteristics and Paxlovid Administration

This study was approved by the Institutional Review Board of the National Cheng Kung University Hospital (NCKUH) (IRB: B-ER-111-164). Written informed consent was obtained from all participants. This study enrolled pregnant women admitted to NCKUH who tested positive for COVID-19 by real-time reverse transcription polymerase chain reaction (RT-PCR) of nasopharyngeal swab specimens. After providing the potential benefits and associated risks of antiviral treatment with Paxlovid, patients made well-informed decisions about whether or not to receive Paxlovid (300/100 mg nirmatrelvir/ritonavir, Pfizer Inc., New York, NY, USA) twice daily for 5 days. Pregnant women were excluded if the time interval between the onset of symptoms and admission to our hospital was greater than seven days, in the case of insufficient saliva samples, or if any antiviral-related agents were administered, such as remdesivir, glucocorticoids, or interleukin-6 receptor antagonists. 

We collected a comprehensive set of medical data, including medical history, physical examination findings, symptom severity, radiological findings, COVID-19 diagnosis and hospitalization date, use of antiviral agents, COVID-19 vaccination status, gestational complications, and gestational age at diagnosis and delivery. The severity of COVID-19 was classified according to the guidelines provided by the American National Institutes of Health [10]. In brief, asymptomatic or presymptomatic infection refers to having no symptoms, while mild illness is characterized by fever, cough, sore throat, malaise, and headache without shortness of breath, dyspnea, or abnormal chest imaging. Moderate illness involved evidence of lower respiratory disease during imaging and oxygen saturation (SpO_2_) of at least 94% in room air at sea level. Severe illness was defined as SpO_2_ < 94% in room air at sea level, PaO_2_/FiO_2_ < 300 mmHg, respiratory rate > 30 breaths/min, or lung infiltration > 50%.

### 2.2. Procedures of Collecting Specimen and PCR

Diagnosis of COVID-19 was confirmed by RT-PCR of nasopharyngeal specimens. After collecting the specimens by inserting a swab along the nasal septum into the nasopharynx, the swabs were placed into a sterile transport tube (Creative Media Plate, Taipei, Taiwan) and directly transferred for RT-PCR. As for saliva specimens, patients were instructed to collect saliva right after admission and until the day of discharge. The saliva was collected under direct supervision with detailed instructions from the posterior oropharynx into a sterile container by coughing or clearing the throat in the early morning prior to brushing their teeth or having a meal. Saliva specimens were stored at −70 °C before analysing. While NP swabs were routinely collected for all participants at admission for diagnostic purposes, saliva sample collection on Day 0 was right after admission, less than one hour after the NP swabs, and was performed only when feasible, resulting in 21 paired samples for comparison on that day.

All nasopharyngeal swabs and saliva specimens underwent processing and SARS-CoV-2 testing at the NCKUH laboratory. Saliva specimens underwent vortexing for one minute, followed by a 30 s incubation at room temperature. The slightly separated supernatant was then removed from a viscous bottom layer, and nucleic acid extraction was performed on the supernatant. Total nucleic acid was extracted from 300 µL of viral transport media from the nasopharyngeal swab and 300 µL of supernatant from saliva using the TANBead Nucleic Acid Extraction Kit (Taiwan Advanced Nanotech Inc., Taoyuan, Taiwan).

For the detection of SARS-CoV-2 RNA, we employed the ABI QuantStudio™ 5 Real-Time PCR System (Thermo Fisher Scientific Inc., Waltham, MA, USA) and SARS-CoV-2 Detection Kit v1 (KimForest, New Taipei City, Taiwan) per the manufacturers’ protocols. Positive identification of SARS-CoV-2 was determined when the cycle threshold (Ct) values were less than 40. A Ct value of more than 40 was interpreted as negative.

### 2.3. Statistics

Statistical analyses were performed using SPSS version 17.0 (IBM Corp., Armonk, NY, USA). Statistical analysis of continuous variables was performed using the Mann–Whitney U test, while categorical variables were analyzed using Fisher’s exact test. The nasopharyngeal and salivary PCR values were compared using the Wilcoxon signed-rank test. Statistical significance was set at 0.05. 

## 3. Results 

### 3.1. Patients Demographics 

**Figure 1:**
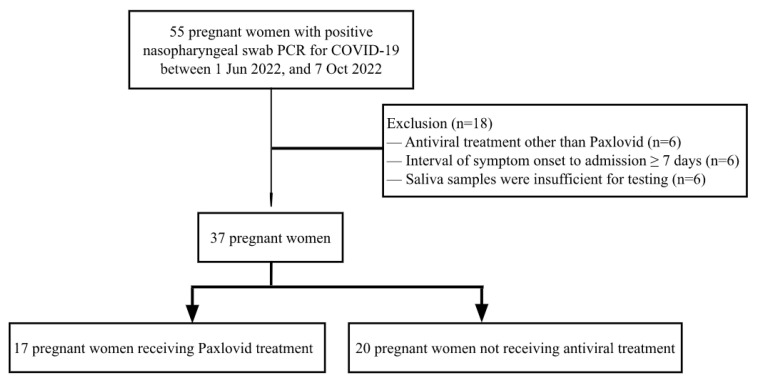
Participant flow diagram. Abbreviation: PCR, polymerase chain reaction; COVID-19, coronavirus disease 2019.

From 1 June 2022 to 7 October 2022, 55 pregnant women were screened for eligibility, among whom 37 were included in the analysis (Figure 1). Of these, 17 pregnant women were administered Paxlovid, and 20 pregnant women were not administered any antiviral treatment. The patient demographics stratified based on the administration of Paxlovid are displayed in Table 1. The two groups of pregnant women were comparable across the two cohorts, except for an earlier gestational age at diagnosis of COVID-19 in the Paxlovid group than in the non-antiviral group (35.4 weeks versus 37.5 weeks, *p* = 0.022). The median age was 32 years. Of the pregnant women, 25.4% received the COVID-19 initial vaccination series only (1 + 2 doses), while 34.5% received the initial vaccination series + Booster dose (3 doses). All of the affected pregnant women were asymptomatic (12 [31.6%]) or had a mild illness (26 [73.7%]). Ten (26.3%) pregnant women experienced gestational complications, including pregnancy-induced hypertension/preeclampsia, gestational diabetes mellitus, antepartum hemorrhage, and preterm birth. Among the women treated with Paxlovid, 47.1% had gestational complications compared with 9.5% of those without antiviral treatment (*p* = 0.012). 

### 3.2. PCR Values from Nasopharyngeal and Saliva Specimens and Change Among Two Groups 

**Figure 2:**
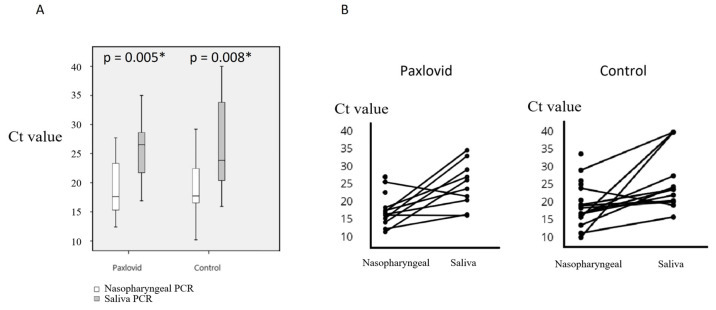
PCR Ct values of pregnant women with COVID-19. (**A**) The histogram illustrates the median and interquartile range of Ct values for pregnant women with COVID-19 in the Paxlovid group and the non-antiviral group. The white bars represent Ct values from nasopharyngeal specimens, while the colored bars represent Ct values from saliva specimens. (**B**) The plot displays individual Ct values from nasopharyngeal and saliva specimens in the Paxlovid group and the non-antiviral group. The values obtained from the nasopharyngeal and saliva specimens within individuals are connected by lines. An asterisk (*) indicates a *p*-value less than 0.05. Abbreviation: PCR, polymerase chain reaction; Ct, cycle threshold; COVID-19, coronavirus disease 2019.

**Figure 3:**
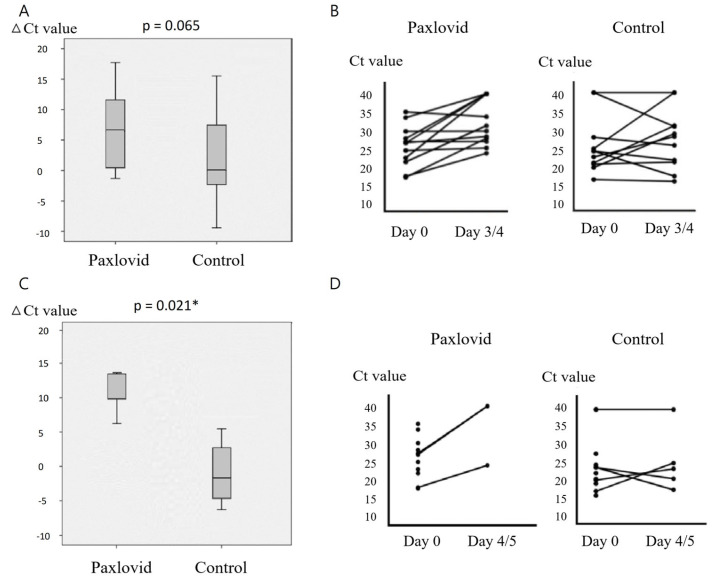
Change in saliva PCR Ct values. (**A**) The histogram illustrates the median and interquartile range of salivary PCR Ct values on Day 3 or Day 4 minus Ct value on Day 0 of the Paxlovid and the non-antiviral groups. (**B**) The plots display individual salivary PCR Ct values on Day 0 and on Day 3 or Day 4 in the Paxlovid and the non-antiviral groups. The Ct values on Day 0 and Day 3 or Day 4 within individuals are connected by lines. (**C**) The histogram illustrates the median and interquartile range of salivary PCR Ct values on Day 4 or Day 5 minus Ct value on Day 0 of the Paxlovid and the non-antiviral groups. (**D**) The plots display individual salivary PCR Ct values on Day 0 and on Day 4 or Day 5 in the Paxlovid and the non-antiviral groups. The Ct values on Day 0 and Day 4 or Day 5 within individuals are connected by lines. An asterisk (*) indicates a *p*-value less than 0.05. Abbreviation: Ct, cycle threshold; PCR, polymerase chain reaction.

The mean duration between diagnosis and treatment initiation was 0.89 days (range: 0–3 days) for the Paxlovid group (Table 1). The Ct values obtained from pre-treatment nasopharyngeal swab specimens for the Paxlovid and non-antiviral groups were comparable, with the median ± interquartile range (IQR) of Ct values being 17.6 ± 9.60 and 17.7 ± 6.73, respectively (*p* = 0.552) (Table 2). There was no significant difference in the salivary Ct values obtained at the time of COVID-19 between the two groups, with the median ± IQR being 26.44 ± 7.68 and 23.78 ± 16.61, respectively (*p* = 0.751) (Table 2). Higher Ct values from saliva specimens were detected among the Paxlovid group, non-antiviral group, and the overall population compared to those from nasopharyngeal swabs before treatment (*p* = 0.005, 0.008, and < 0.001, respectively) (Figure 2). Since previous studies [2,4] have shown that viral load peaked early and then gradually declined under treatment, we compared the change in salivary PCR Ct values (Ct value on Day 3 or Day 4 minus on Day 0, and Ct value on Day 4 or Day 5 minus on Day 0) among the two groups. We intended to choose the Ct value on day 3 and day 5 for evaluating the change in salivary PCR Ct values, but data on day 4 would be used to evaluate the trend of Ct value when there were no data available on day 3 or day 5. The results showed that the change in relative viral loads was higher in the Paxlovid group compared to the non-antiviral group, with a median ± IQR of 6.48 ± 11.75 versus 0.00 ± 11.83 (*p* value = 0.065) and 13.40 ± 5.64 versus −1.59 ± 9.63 (*p* = 0.021), respectively (Table 2) (Figure 3). 

### 3.3. Detection Rate and Salivary PCR Ct Values of Patients Among the Two Groups

Both the Paxlovid and non-antiviral groups had 12 saliva specimens collected on the day of COVID-19 diagnosis (Day 0). Fifteen pregnant women in the Paxlovid group self-collected saliva specimens for more than three consecutive days, and 17 pregnant women in the non-antiviral group completed a consecutive collection of specimens. (Table 3 and Table 4). In the Paxlovid group, six pregnant women showed an increase in salivary PCR Ct values, five showed a decrease followed by an increase in salivary PCR Ct values, two showed a decrease in salivary PCR Ct values, and two showed salivary PCR Ct values below the limit of detection throughout the surveillance period (Table 3). In the non-antiviral group, four pregnant women showed an increase in salivary PCR Ct values, two showed a decrease followed by an increase in salivary PCR Ct values, nine showed fluctuating salivary PCR Ct values, and two showed salivary PCR Ct values below the limit of detection across all tests (Table 4). The detection rate of COVID-19 using salivary PCR was 83.8%, with 31 patients testing positive out of the 38 patients diagnosed using nasopharyngeal swabs. 

## 4. Discussion

In this study, salivary PCR was investigated for its application as a diagnostic tool for COVID-19 and the antiviral effect of Paxlovid in pregnant women. We found significantly higher Ct values in COVID-19 pregnant women from saliva specimens than from nasopharyngeal ones. In addition, Paxlovid showed an effective antiviral effect on viral load reduction from salivary PCR. Compared with nasopharyngeal PCR, salivary PCR had a detection rate of 83.8% in pregnant women. These results suggested that self-collected saliva is a useful diagnostic tool for COVID-19 pregnant women. 

A previous study demonstrated that the average Ct value for SARS-CoV-2 in saliva samples was 9.3 times higher compared to that in nasopharyngeal swabs [11]. Our findings are consistent as we observed Ct values that were 8.84 and 6.08 higher in saliva specimens than in nasopharyngeal specimens in the antiviral and non-antiviral groups, respectively. Similarly, a systemic review comparing the performance of nucleic acid amplification testing using various specimen sources concluded that saliva samples had a lower diagnostic performance than nasopharyngeal swabs [12]. Nevertheless, some studies have indicated that saliva samples exhibit higher sensitivity compared to nasopharyngeal swabs in the early stage of symptom onset [13]. Furthermore, a study also revealed contradictory results showing that the salivary PCR Ct value was 8.3 times lower than that of nasopharyngeal specimens [14]. 

Nasopharyngeal swabs have proven to be an efficient way to detect respiratory viruses and are the most widely used method for COVID-19 diagnosis. Nevertheless, nasopharyngeal swabs require specialized expertise to collect the specimens properly. In addition, the process of obtaining specimens may lead to concerns about the spread of respiratory viruses. Saliva has been demonstrated to be a promising modality for frequent and repeated testing to reduce the risk of transmission. A previous study showed that the positivity rate of saliva testing ranged from 80.4% to 86.5% [15]. Patients presenting with COVID-19-associated symptoms had a greater likelihood of testing positive by salivary PCR within the first week of infection compared to those without symptoms (88.2% vs. 58.2%; odds ratio 2.84, *p* < 0.001) [16]. Likewise, in our study, the salivary PCR detection rate within 1 week of symptom onset was 92.3% (24/26) in symptomatic pregnant women and 58.3% (7/12) in asymptomatic participants. The overall detection rate of SARS-CoV-2 using salivary PCR of 83.8% compared to nasopharyngeal PCR in our study was consistent with previous research [11]. Even though it has been suggested that saliva specimens could serve as an attractive alternative, especially in an ambulatory setting due to reduced patient discomfort and easier sample retrieval [17].

Nirmatrelvir/ritonavir (Paxlovid) inhibits the main protease of SARS-CoV-2, reducing viral load and preventing the assembly of new virions, which can effectively decrease the progression to severe disease and quickly reduce SARS-CoV-2 viral load when administered early in COVID-19 illness [18]. Specifically, Paxlovid reduced viral load by a factor of 10 relative to placebo when treatment was initiated within 3 days after symptom onset [4]. Our results demonstrate that a 5-day course of Paxlovid as recommended, initiated early after diagnosis, is associated with a significant reduction in SARS-CoV-2 viral load in pregnant women compared to no antiviral treatment. In addition, Paxlovid reduced the time to achieve low viral burden, with a significant increase in the Ct value between baseline and days 5–7 compared to the control group [19]. There had been scarce reports adopting salivary viral load for assessing the treatment efficacy of Paxlovid. Considering the potential fluctuation of viral load throughout the duration of COVID-19 infection and drawing from prior research on nasopharyngeal specimens indicating a downward trend during antiviral treatment, our study evaluated the efficacy of antiviral agents through the analysis of changes in salivary PCR Ct values between day 3/day 4 or day 4/day 5 and the day of COVID-19 diagnosis. Our study revealed a decrease in salivary viral load within the antiviral-treated group as opposed to the group that did not receive antiviral agents. These findings reinforce the necessity of taking Paxlovid for a continuous period of 5 days. 

Women treated with Paxlovid in the current study tended to have a lower gestational age at diagnosis and a higher likelihood of gestational complications than women not treated with antivirals. These findings may be a result of selection bias since most of these conditions pre-existed before the commencement of Paxlovid treatment. Real-world use of Paxlovid still warrants further studies to evaluate its long-term safety as concerns of cesarean delivery and small for gestational age may exist [20]. A recent target trial emulation enrolling 211 pregnant women receiving Paxlovid and 1998 matched controls for analysis demonstrated that Paxlovid was associated with reduced maternal morbidity, cesarean section, and preterm birth [21]. However, the incidence of pre-existing obstetric complications, such as hypertensive disorders of pregnancy and gestational diabetes mellitus, was higher in our study group compared to this study. This disparity may have significant implications for pregnancy outcomes. Therefore, further research is needed to investigate the efficacy and safety of Paxlovid in pregnant women and to identify the subgroups that would derive the greatest benefits from antiviral use.

Our prospective study has several limitations. First, the quality of pregnant women’s self-collected oropharyngeal saliva samples may not be consistent. Although the women were instructed to collect deep oropharyngeal saliva samples, it was difficult to ensure that they coughed up the deep pharyngeal saliva. Second, some pregnant women did not provide sufficient consecutive saliva samples, which hindered the data analysis. Third, our study was limited by the inclusion of only asymptomatic and mild COVID-19 cases. As such, we could not assess the impact of disease severity on Ct values. Future studies with a broader range of disease severity are needed to investigate this potential relationship. This would provide a more comprehensive understanding of the dynamics of viral load in relation to clinical presentation. Fourth, paired NP and saliva samples were not available for all participants on Day 0. This limits our ability to make definitive conclusions about the relative sensitivity of salivary PCR compared to the gold standard NP swab in our specific cohort. The observed difference in Ct values between saliva and NP samples might be influenced by the non-random selection of participants who provided both samples. Finally, the limited sample size highlights the need for future studies with larger populations to validate the diagnostic value of salivary PCR in pregnant women with COVID-19.

Our results demonstrate that self-collected saliva is a useful diagnostic tool for SARS-CoV-2 infection in pregnant women. A significant decrease in the relative viral load from saliva specimens was observed among pregnant women treated with Paxlovid compared to women not treated with antiviral agents. Whether this finding fits with other vulnerable or immune-comprised populations requires validation in further studies. Also, given our limited sample size and the potential for confounding factors to influence the time to delivery, we have noted this as an important area for future research.

## Figures and Tables

**Table 1:** Demographic and clinical characteristics of the pregnant women affected with COVID-19.

	Pregnant Women with Paxlovid Treatment (*n* = 17)	Pregnant Women Without Antiviral (*n* = 20)	*p* Value
Age, mean ± SD, years	32.82 ± 5.7	31.8 ± 4.2	0.479
BMI, mean ± SD, kg/m^2^	25.96 ± 4.2	25.7 ± 3.2	0.747
Gestational age at COVID-19 infection —weeks, mean ± SD, weeks (range)	35.4 ± 4.5 (22~39.3)	37.5 ± 3.1 (27.9~40.4)	0.022 *
Gestational age at delivery— weeks, mean ± SD, weeks (range)	37.0 ± 3.7 (24.1~40)	38.8 ± 1.2 (31.6~41.3)	0.002 *
Interval of diagnosis to treatment, mean ± SD, days (range)	0.89 ± 0.92 (0~3)	-	-
Interval of diagnosis to saliva collection, mean ± SD, days (range)	1.3 ± 1.6 (0~5)	1.8 ± 1.9 (0~5)	0.464
COVID-19 vaccination —no. of pregnant women (%)			
Not received	2 (11.8%)	3 (15%)	1.000
Initial vaccination series only (1 + 2 doses)	7 (41.2%)	7 (35%)	0.740
Initial vaccination series + Booster dose (3 doses)	8 (47%)	10 (50%)	1.000
COVID-19 severity —number of pregnant women (%)			
Asymptomatic	3 (17.6%)	9 (45%)	0.161
Mild illness	14 (82.4%)	11 (55%)	0.161
Moderate and severe illness	0 (0%)	0 (0%)	-
Gestational complications —number of pregnant women (%)	8 (47.1%)	2 (10%)	0.012 *
Pregnancy induced hypertension/preeclampsia	2 (11.8%)	0 (0%)	0.193
Gestational diabetes mellitus	2 (11.8%)	1 (5%)	0.419
Antepartum hemorrhage	1 (5.9%)	1 (5%)	0.701
Preterm birth	4 (23.5%)	0 (0%)	0.032 *

Abbreviation: COVID-19: coronavirus disease 2019, SD: standard deviation, BMI: body mass index. * *p* < 0.05.

**Table 2:** PCR Ct values of pregnant women with COVID-19.

	Pregnant Women with Paxlovid Treatment (*n* = 17)	Pregnant Women Without Antiviral Treatment (*n* = 20)	*p*-Value
Pre-medication nasopharyngeal PCR Ct value (Day 0), median ± IQR	17.60 ± 9.60	17.70 ± 6.730	0.552
Salivary PCR Ct value at the day of diagnosis with COVID-19 (Day 0), median ± IQR	26.44 ± 7.68 *n* = 12	23.78 ± 16.61 *n* = 12	0.751
Salivary PCR Ct value change (Ct value on Day 3 or Day 4 minus Day 0), median ± IQR	6.48 ± 11.75 *n* = 12	0.00 ± 11.83 *n* = 11	0.065
Salivary PCR Ct value change (Ct value on Day 4 or Day 5 minus Day 0), median ± IQR	13.40 ± 5.64 *n* = 4	−1.59 ± 9.63 *n* = 4	0.021 *

Abbreviation: COVID-19: coronavirus disease 2019; Ct, cycle threshold; IQR, interquartile range; PCR, polymerase chain reaction. * *p* < 0.05.

**Table 3:** Pregnant women with COVID-19 receiving Paxlovid treatment.

No.	Age (Years)	BMI (kg/m^2^)	Gestational Age at Diagnosis (Weeks)	Gestational Age at Delivery (Weeks)	Delivery Method	Pre-Medication Nasopharyngeal PCR Ct Value (Day 0)	Salivary PCR Ct Value								Change in Salivary PCR Ct Value		Vaccination Dose			COVID-19 Disease Severity		Gestational Complications and Medical Comorbidities
							Day 0 (Diagnosis Day)	Day 1	Day 2	Day 3	Day 4	Day 5	Day 6	Day 7	Δ (Day 3 or Day 4–Day 0)	Δ (Day 4 or Day 5–Day 0)	1st Dose	2nd Dose	1st Booster	Asymptomatic	Mild	
P1	37	28.13	34.7	36.0	C/S	13.4	26.4	30.92	Neg	Neg	Neg	Neg			13.6	13.6					✓	Twin pregnancy, Preterm birth
P2	26	38.09	37.7	37.7	C/S	15.3	26.47	23.42	-	28.14	Neg				1.67	13.53	✓	✓			✓	PIH
P3	32	28.89	36.1	36.9	C/S	27.7						Neg	Neg	Neg						✓		Preterm birth
P4	36	27.25	31.4	38.9	C/S	23.3					20.78	21.25	20.43				✓	✓			✓	
P5	36	27.58	38.9	39.1	C/S	13.2	17.17	26.84	Neg	23.52	22.22	23.34	28.99		6.35	6.17	✓	✓	✓		✓	GDM, Thyroid disease
P6	30	25.77	31.9	N/A ^a^	N/A ^a^	26.2	22.25	20.48	34.57	Neg					17.75		✓	✓	✓		✓	GDM
P7	38	21.99	22.0	24.1	VD	12.4	26.73	27.52	23.22	26.81	37.7	Neg	Neg		0.08	13.27	✓	✓	✓		✓	Placenta previa, APH, Preterm birth
P8	28	24.68	38.7	39.0	VD	26.2				Neg	Neg						✓	✓	✓	✓		PIH
P9	40	30.80	35.1	36.1	VD	17.2	21.15	21.82	27.09	31.2					10.1		✓	✓			✓	Preterm birth
P10	37	25.97	39.3	39.4	VD	16.1	33.4	Neg	Neg	Neg					6.6		✓	✓	✓		✓	
P11	21	25.08	37.7	37.7	VD	15	29.6		17.7	29.7					0.1		✓	✓			✓	
P12	38	23.83	38.3	38.3	C/S	18.3	24.3		29.1	25					0.7		✓	✓	✓		✓	
P13	26	22.66	37.3	37.3	VD	19.1	27.6	26.4	Neg	Neg					12.4		✓	✓		✓		
P14	38	20.51	37.4	37.6	VD	18	35		33.6	33.7					−1.3		✓	✓	✓		✓	
P15	30	23.53	29.6	N/A ^a^	N/A ^a^	17	16.9		22.2	27.7					10.8		✓	✓	✓		✓	
P16	27	21.88	38.4	38.9	VD	17.6				Neg	Neg	26.9					✓	✓			✓	
P17	38	24.61	38.0	38.0	VD	27.5		32.9	28.5								✓	✓			✓	

Note: Gray-colored grid denotes prescription day of Paxlovid, Day 0 denotes the diagnosis day of COVID-19 infection by nasopharyngeal swab; viral loads below the limit of detection are labeled as Neg; N/A, not available; ^a^ Yet to deliver during the period of surveillance; ✓, vaccination was done; -, data not available. Abbreviation: COVID-19, coronavirus disease 2019; BMI, body mass index; PCR, polymerase chain reaction; Ct, cycle threshold; C/S, cesarean section; PIH, pregnancy-induced hypertension; GDM, gestational diabetes mellitus; VD, vaginal delivery; APH, antepartum hemorrhage.

**Table 4:** Pregnant women with COVID-19 receiving no antiviral treatment.

No.	Age (Years)	BMI (kg/m^2^)	Gestational Age at Diagnosis (Weeks)	Gestational Age at Delivery (Weeks)	Delivery Method	Pre-Medication Nasopharyngeal PCR Ct Value (Day 0)	Salivary PCR Ct Value								Change in Salivary PCR Ct Value		Vaccination Dose			COVID-19 Disease Severity		Gestational Complications and Medical Comorbidities
							Day 0 (Diagnosis Day)	Day 1	Day 2	Day 3	Day 4	Day 5	Day 6	Day 7	Δ (Day 3 or Day 4–Day 0)	Δ (Day 4 or Day 5–Day 0)	1st Dose	2nd Dose	1st Booster Dose	Asymptomatic	Mild	
C1	34	22.64	38.1	38.3	VD	16.9				27.31	33.6						✓	✓			✓	Thyroid disease
C2	32	19.57	37.9	38.0	C/S	19.2	20.55	24.95	30.6	30.86					10.3						✓	
C3	34	26.77	38.4	38.9	VD	29.2	Neg	Neg	Neg	Neg	Neg				0	0	✓	✓			✓	
C4	29	26.37	39.6	40.0	VD	16.6				31.78	Neg	26.93	Neg				✓			✓		
C5	42	29.68	35.1	38.4	C/S	16.6	27.6	26.76	31.96	25.41					−2.19						✓	GDM
C6	23	25.78	40.4	41.3	VD	20.7						20.64	33.07				✓	✓	✓		✓	
C7	32	28.49	38.9	39.6	VD	18.7						26.96	30.11	30.72			✓				✓	
C8	31	25.15	38.9	39.4	VD	26.2					32.04	Neg	37.98	Neg			✓	✓	✓		✓	
C9	25	23.83	39.4	39.6	VD	16.9	23.78	21.09	14.03	16.87	17.51				−6.91	−6.27	✓	✓	✓	✓		PPH
C10	30	25.95	38.0	39.7	VD	16.5				18.7	17.66	21.08	17.44				✓	✓	✓	✓		PPH
C11	32	24.80	38.1	38.7	VD	25.2						35.74	36.3				✓	✓	✓		✓	
C12	28	32.74	38.0	39.0	VD	24.1	19.27	25.12	22.79	28.62					9.35		✓	✓	✓	✓		
C13	32	22.54	38.6	39.1	C/S	N/A ^a^				17.12	15.83	21.99	22.7	25			✓	✓	✓		✓	
C14	34	23.23	38.9	39.0	VD	19.4	23.78	18.72	17.27	21.3	20.61				−2.48	−3.17	✓	✓		✓		
C15	32	27.73	36.9	38.7	VD	33.8		Neg	Neg	Neg							✓	✓	✓	✓		APH
C16	35	29.48	27.9	N/A ^b^	N/A ^b^	18.5	20.2	28.77	23.93	20.75	23.37	25.71			0.55	5.51	✓	✓			✓	
C17	30	22.21	39.7	39.9	VD	16.8	22.15		26.11	27.81					5.66					✓		
C18	31	29.05	38.0	38.0	VD	13.6	24.47	31.56	31.7	Neg					15.53		✓	✓			✓	
C19	31	21.39	29.9	N/A ^b^	N/A ^b^	11.4	15.91	12.41	16.54	15.43					−0.48		✓	✓	✓		✓	
C20	40	25.20	38.9	38.9	VD	15.9	Neg	Neg									✓	✓	✓	✓		
C21	31	28.23	38.6	38.6	C/S	10.2	Neg	Neg	Neg	30.6					−9.4		✓	✓	✓	✓		

Note: Day 0 denotes the diagnosis day of COVID-19 by nasopharyngeal swab; viral loads below the limit of detection are labeled as Neg. ^a^ No nasopharyngeal PCR Ct value available. ^b^ Yet to deliver during the period of surveillance; N/A, not available; ✓, vaccination was done;. Abbreviation: COVID-19, coronavirus disease 2019; BMI, body mass index; PCR, polymerase chain reaction; Ct, cycle threshold; VD, vaginal delivery; C/S, cesarean section; GDM, gestational diabetes mellitus; PPH, postpartum hemorrhage; APH, antepartum hemorrhage.

## Data Availability

The original contributions presented in this study are included in the article. Further inquiries can be directed to the corresponding authors.
